# The AURKA-Selective Inhibitor Alisertib Attenuates Doxorubicin-Induced Hepatotoxicity in Mice via Modulation of IL-17A/NF-κB and STAT3 Signaling Pathways

**DOI:** 10.3390/ph18081201

**Published:** 2025-08-14

**Authors:** Faisal Alqussair, Mahmoud Elshal, Mirhan N. Makled, Nashwa M. Abu-Elsaad

**Affiliations:** Department of Pharmacology and Toxicology, Faculty of Pharmacy, Mansoura University, El Gomhoria Street, Mansoura 35516, Eldakahlia, Egypt

**Keywords:** Aurora kinase, alisertib, doxorubicin, hepatotoxicity, fibrosis

## Abstract

**Background/Objectives:** Doxorubicin (DOXO) is effective against various types of cancer; however, it is associated with hepatotoxicity that may eventually lead to liver fibrosis, limiting its clinical use. Aurora kinase A (AURKA) has emerged as a crucial regulator of essential cellular processes and a promising target to overcome tumors resistant to some anticancer drugs, including DOXO. However, the potential beneficial effect of targeting AURKA on DOXO-induced toxicities has not been explored yet. Therefore, the current study aimed to explore the potential protective effect of the AURKA-selective inhibitor alisertib on DOXO-induced hepatotoxicity in mice and address the possible underlying mechanism. **Methods:** Mice were treated with alisertib (10 and 20 mg/kg) daily for five consecutive days and challenged with DOXO (20 mg/kg, i.p.) once on day two. **Results:** Our findings revealed that alisertib significantly reduced biomarkers of liver dysfunction and oxidative stress elevated by the DOXO challenge. Interestingly, alisertib suppressed DOXO-induced IL-17A upsurge along with NF-κB and STAT3 activation. Alisertib also suppressed the upregulated expression of HIF-1α and VEGF-A as well as PERK activation associated with the DOXO challenge. Moreover, alisertib counteracted DOXO-induced TGF-β1 and α-SMA overexpression in the liver. These beneficial effects of alisertib were further reflected in the histopathological findings, which indicated the ability of alisertib to ameliorate DOXO-induced hepatic necroinflammation and fibrosis. **Conclusions:** Alisertib mitigates DOXO-induced hepatotoxicity in mice via targeting the IL-17A/NF-κB and IL-17A/STAT3/HIF-1α/VEGF-A signaling pathways, attenuating oxidative stress, inflammation, ER stress, and fibrosis.

## 1. Introduction

Drug-induced liver injury is a harmful toxic reaction caused by many drugs and can lead to liver damage. Among these drugs is doxorubicin (DOXO), which is an antineoplastic antibiotic that belongs to the anthracycline class. DOXO is effective against various types of cancer, such as breast cancer, bladder cancer, and leukemia. It exerts its anticancer effect by intercalating DNA, inhibiting topoisomerase II, and producing reactive oxygen species (ROS), which cause DNA double-strand breaks and cell cycle arrest in rapidly dividing tumor cells [1,2].

DOXO is associated with crucial toxicities like cardiotoxicity and hepatotoxicity, which may limit its clinical use [3]. Its hepatotoxic effects are mainly mediated by excessive ROS generation, which enhances lipid peroxidation, mitochondrial dysfunction, DNA damage, and pro-inflammatory pathway activation, including the NF-κB and STAT3 signaling pathways, pro-inflammatory cytokine release, and subsequent fibrotic remodeling. However, the exact mechanisms underlying DOXO-induced hepatotoxicity are still incompletely understood [4,5,6,7]. Recently, different studies have demonstrated the multifactorial nature of drug-induced liver injury, including complex interactions between oxidative stress, inflammatory mediators, and fibrogenic pathways [8,9]. These findings highlight the need for targeted therapies that modulate both oxidative and inflammatory networks to counteract chemotherapy-induced liver damage.

Serine/threonine kinases are enzymes that transfer phosphate from ATP to a protein substrate, specifically on a serine or threonine amino acid residue. The Aurora family of serine/threonine kinases has emerged as crucial regulators of essential processes ranging from mitotic entry to cytokinesis. Dysregulated Aurora kinase A (AURKA) has been shown to mediate various cancer types [10,11]. AURKA is known to control a number of downstream signaling cascades that affect cell proliferation, survival, and stress responses, such as NF-κB and STAT3 activation [12,13].

Alisertib is an investigational AURKA-selective inhibitor that demonstrated efficacy against various tumor types in both in vitro and in vivo settings [14]. Recently, AURKA has been reported to play an important role in some anticancer resistance, where alisertib enhanced the antitumor activity of cisplatin and paclitaxel in vitro in platinum-resistant high-grade serous ovarian cancer [15]. Inhibiting AURKA increased breast cancer sensitivity towards DOXO in vitro [16]. Additionally, silencing AURKA boosted the activity of cyclophosphamide, DOXO, vincristine, and prednisone in diffuse large B-cell lymphoma [17]. However, the potential role of AURKA in anticancer-associated toxicities has not been explored yet. We hypothesize that AURKA may represent a promising target for decreasing some anticancer-associated toxicity as well.

Given that DOXO-induced hepatotoxicity involves ROS-driven pro-inflammatory signaling and considering AURKA’s regulatory role in NF-κB and STAT3 signaling, we hypothesized that inhibiting AURKA might attenuate DOXO-induced liver damage. Accordingly, this study aimed to investigate the potential protective role of alisertib against DOXO-induced hepatotoxicity in mice and explore its underlying molecular mechanisms.

## 2. Results

### 2.1. Effect of Alisertib on DOXO-Induced Hepatocellular Injury

**Figure 1:**
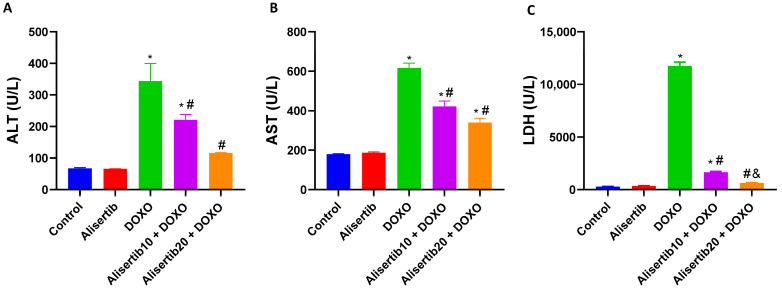
Effect of alisertib (10 and 20 mg/kg) on serum levels of (**A**) alanine aminotransferase (ALT), (**B**) aspartate aminotransferase (AST), and (**C**) lactate dehydrogenase (LDH) in doxorubicin (DOXO)-intoxicated mice. Data are expressed as means ± SEs (n = 6). *, #, and & indicate significant differences compared to the control, DOXO, and alisertib10 + DOXO groups, respectively, using one-way ANOVA followed by the Tukey–Kramer multiple-comparisons post hoc test.

Hepatocellular injury after the DOXO challenge was evidenced by a significant (*p* < 0.001) increase in ALT, AST, and LDH serum activities in comparison with control mice (Figure 1A–C, respectively). However, the administration of alisertib (10 mg/kg) for 5 days with DOXO significantly decreased peak activities of ALT (*p* < 0.05), AST (*p* < 0.001) and LDH (*p* < 0.001), compared to DOXO alone. Otherwise, a higher alisertib dose (20 mg/kg) significantly decreased peak activities of ALT (*p* < 0.001), AST (*p* < 0.001), and LDH (*p* < 0.001), compared to DOXO alone. Notably, a significant difference (*p* < 0.01) was observed between the two tested doses in LDH activity, which was lower in the alisertib20 + DOXO group.

### 2.2. Effect of Alisertib on DOXO-Induced Hepatic Oxidative Stress

**Figure 2:**
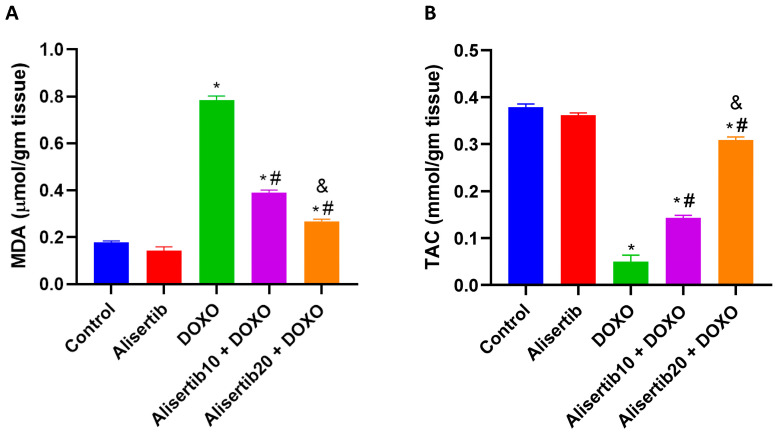
Effect of alisertib (10 and 20 mg/kg) on hepatic levels of (**A**) malondialdehyde (MDA) and (**B**) total antioxidant capacity (TAC) in doxorubicin (DOXO)-intoxicated mice. Data are expressed as means ± SEs (n = 6). *, #, and & indicate significant differences compared to the control, DOXO, and alisertib10 + DOXO groups, respectively, using one-way ANOVA followed by the Tukey–Kramer multiple-comparisons post hoc test.

DOXO injection resulted in redox imbalance in the liver, as indicated by a significant (*p* < 0.001) increase in the lipid peroxidation biomarker MDA (by about 4.4-fold) and a decrease in hepatic TAC (by about 7.7-fold), compared to the control group (Figure 2A and Figure 2B, respectively). Meanwhile, alisertib (10 and 20 mg/kg) significantly (*p* < 0.001) repressed hepatic MDA contents by about 50.2% and 65.9%, respectively, compared to the DOXO group. Alternatively, hepatic TAC levels were significantly (*p* < 0.001) boosted upon treatment with alisertib (10 and 20 mg/kg) by about 189.9% and 534.7%, respectively, compared to the DOXO group. Notably, the effect of alisertib (20 mg/kg) on MDA and TAC in the liver was significantly (*p* < 0.001) superior compared to the effect of alisertib (10 mg/kg).

### 2.3. Effect of Alisertib on DOXO-Induced Hepatic Histopathological Abnormalities

**Figure 3:**
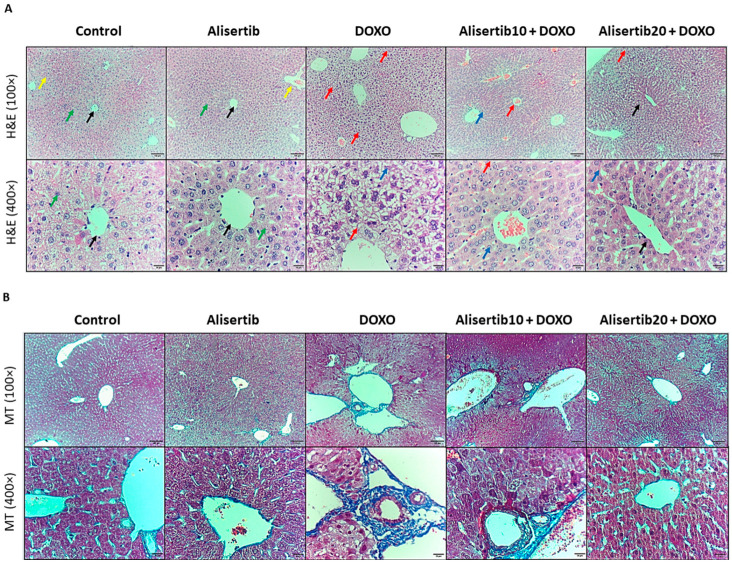

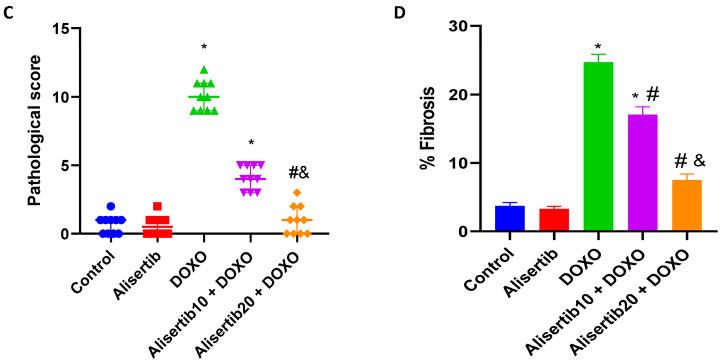
(**A**) Representative micrographs of hematoxylin-and-eosin (H&E)-stained liver tissue sections demonstrating the effect of alisertib (10 and 20 mg/kg) on doxorubicin (DOXO)-induced hepatic histopathological lesions in mice (upper panel: ×10, bar = 100 µm; lower panel: ×40, bar = 20 µm). The control and alisertib groups show normal liver architecture. The DOXO group shows central vein and portal triad congestion and dilatation, increased Kupffer cell activity, and hepatocyte vacuolation and necrosis. The alisertib10 + DOXO group shows mild central vein and portal triad congestion with minimal dilatation, increased Kupffer cell activity, and minimal hepatocyte necrosis. The alisertib20 + DOXO group shows minimal central vein dilatation with restoration of normal architecture. Green arrows: hepatocytes, black arrows: central veins, yellow arrows: portal triads, blue arrows: hepatocyte vacuolation or necrosis, and red arrows: congested or dilated central veins and portal triads. (**B**) Representative micrographs of Masson’s trichrome (MT)-stained liver tissue sections demonstrating the effect of alisertib on DOXO-induced hepatic fibrotic changes in mice (upper panel: ×10, bar = 100 µm; lower panel: ×40, bar = 20 µm). The control and alisertib groups show negative to minimal fibrosis in the walls of central veins, portal triads, and blood sinusoids. The DOXO group shows high fibrosis and bridging between the portal triads and central veins. Alternatively, the alisertib10 + DOXO group shows mild fibrosis in the walls of the portal triads with minimal fibrosis in the walls of central veins. The alisertib20 + DOXO group shows minimal fibrosis in the walls of the portal triads and central veins. (**C**) Scatter dot plots of the corresponding pathological scores. Data are expressed as medians and interquartile ranges. *, #, and & indicate significant differences compared to the control, DOXO, and alisertib10 + DOXO groups, respectively, using the Kruskal–Wallis test followed by Dunn’s tests for pairwise comparisons. (**D**) Corresponding percentages of fibrosis. Data are expressed as means ± SEs. *, #, and & indicate significant differences compared to the control, DOXO, and alisertib10 + DOXO groups, respectively, using one-way ANOVA followed by the Tukey–Kramer multiple-comparisons post hoc test.

Liver tissue sections from the experimental groups were stained with H&E and MT stains to address necroinflammatory and fibrotic lesions, represented in Figure 3A and Figure 3B, respectively. Our results showed that liver sections from the DOXO group showed central vein and portal triad congestion and dilatation, increased Kupffer cell activity, and hepatocyte vacuolation and necrosis, in addition to high fibrosis and bridging between the portal triads and central veins when compared to the control group. However, these hepatic pathological abnormalities were attenuated in the alisertib10 + DOXO group, demonstrating mild central vein and portal triad congestion and dilatation, minimal activity of Kupffer cells, and minimal hepatocyte necrosis, along with mild fibrosis in the walls of the portal triads with minimal fibrosis in the walls of central veins. Otherwise, treatment with alisertib (20 mg/kg) with DOXO resulted in minimal central vein dilatation with restoration of normal hepatic architecture, as well as minimal fibrosis in the walls of the portal triads and central veins.

These necroinflammatory changes were scored, and the fibrotic percentages were determined for further illustration (Figure 3C and Figure 3D, respectively). Our data revealed a significantly (*p* < 0.001) greater pathological score and percentage of fibrosis in the DOXO group when compared to the control counterpart. However, treatment with alisertib (10 mg/kg) with DOXO reduced the pathological score (by about 59.4%) and percentage of fibrosis (by about 31.1%) compared to DOXO alone. Otherwise, a higher dose of alisertib (20 mg/kg) showed a significantly superior effect to the lower dose and reduced the pathological score (by about 90.1%) and the percentage of fibrosis (by about 69.7%) compared to DOXO alone.

Notably, the alisertib group did not show any significant difference compared to the control regarding all histopathological changes as well as hepatocellular injury and oxidative stress biomarkers. Accordingly, we excluded this group from the subsequent molecular measures.

### 2.4. Effect of Alisertib on DOXO-Induced Liver Activation of NF-κB

**Figure 4:**
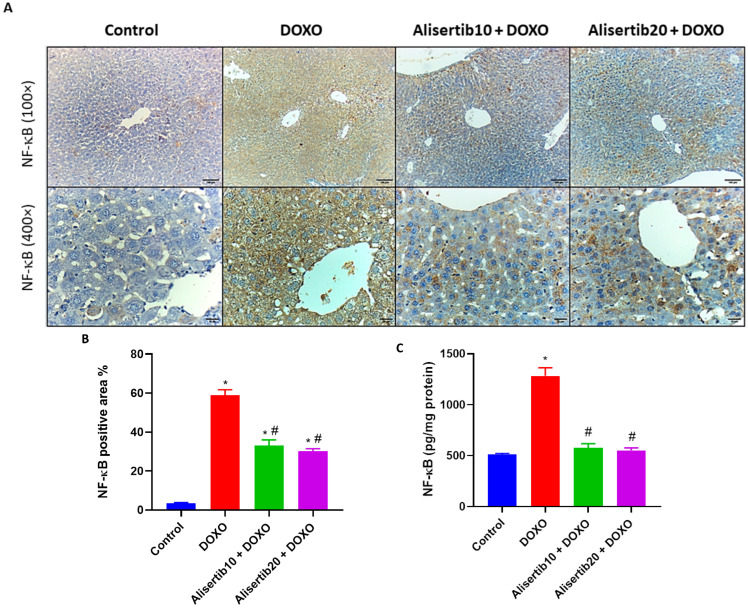
(**A**) Representative micrographs of immuno-stained liver tissue sections demonstrating the effect of alisertib (10 and 20 mg/kg) on nuclear factor kappa B (NF-κB) expression in doxorubicin (DOXO)-intoxicated mice (upper panel: ×10, bar = 100 µm; lower panel: ×40, bar = 20 µm). The control group shows normal hepatic architecture with minimal NF-κB immunoreactivity in a few hepatocytes. The DOXO group shows intense immunoreactivity in all hepatocytes. The alisertib10 + DOXO group shows moderate immunoreactivity in all hepatocytes. The alisertib10 + DOXO group shows mild immunoreactivity in most of the hepatocytes. (**B**) Corresponding percentages of NF-κB immunoexpression-positive areas. (**C**) Effect of alisertib (10 and 20 mg/kg) on hepatic levels of NF-κB in DOXO-intoxicated mice. Data are expressed as means ± SEs. * and # indicate significant differences compared to the control and DOXO groups, respectively, using one-way ANOVA followed by the Tukey–Kramer multiple-comparisons post hoc test.

Immunohistochemical assessment of the transcription factor NF-κB p65 revealed that the DOXO challenge strongly elevated its expression in all hepatocytes compared to its minimal expression in a few hepatocytes in the control group (Figure 4A). Alternatively, the alisertib10 + DOXO group showed moderate NF-κB p65 expression in all hepatocytes, which was further repressed in the alisertib20 + DOXO group that demonstrated mild NF-κB p65 expression in most of the hepatocytes. For further illustration, the percentage of NF-κB p65-positive area was determined, and it was demonstrated that NF-κB p65 immunoexpression was significantly (*p* < 0.001) elevated in the DOXO group by about 16.7-fold, compared to the control group (Figure 4B). Otherwise, treatment with alisertib (10 and 20 mg/kg) with DOXO significantly (*p* < 0.001) suppressed this expression by about 43.8% and 48.6%, respectively, compared to DOXO alone.

For further confirmation, the effect of alisertib on NF-κB p65 was also addressed by ELISA in liver homogenates. As shown in Figure 4C, DOXO significantly (*p* < 0.001) elevated hepatic NF-κB p65 expression compared to the control group. Meanwhile, both doses of alisertib significantly (*p* < 0.001) reduced its expression, compared to the DOXO group.

### 2.5. Effect of Alisertib on DOXO-Induced Elevation of IL-17A, TGF-β1, and α-SMA Hepatic Expression

**Figure 5:**
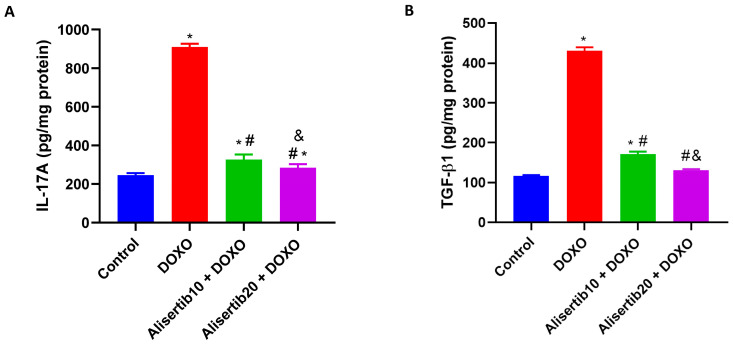
Effect of alisertib (10 and 20 mg/kg) on hepatic levels of (**A**) interleukin (IL)-17A and (**B**) transforming growth factor (TGF) β1 in doxorubicin (DOXO)-intoxicated mice. Data are expressed as means ± SEs (n = 6). *, #, and & indicate significant differences compared to the control, DOXO, and alisertib10 + DOXO groups, respectively, using one-way ANOVA followed by the Tukey–Kramer multiple-comparisons post hoc test.

When compared to the control group, DOXO injection significantly (*p* < 0.001) elevated the levels of pro-inflammatory IL-17A (by about 3.7-fold) and pro-fibrotic TGF-β1 (by about 3.6-fold) in the liver (Figure 5A and Figure 5B, respectively). Meanwhile, alisertib administration (10 and 20 mg/kg) with DOXO significantly reduced the elevated levels of IL-17A (by about 64.1% and 68.7%, respectively) and TGF-β1 (by about 60.2% and 69.5%, respectively) in comparison to the DOXO group. Notably, the effect of the higher dose of alisertib was significantly higher than that of the lower dose on both IL-17A and TGF-β1 hepatic expression levels.

**Figure 6:**
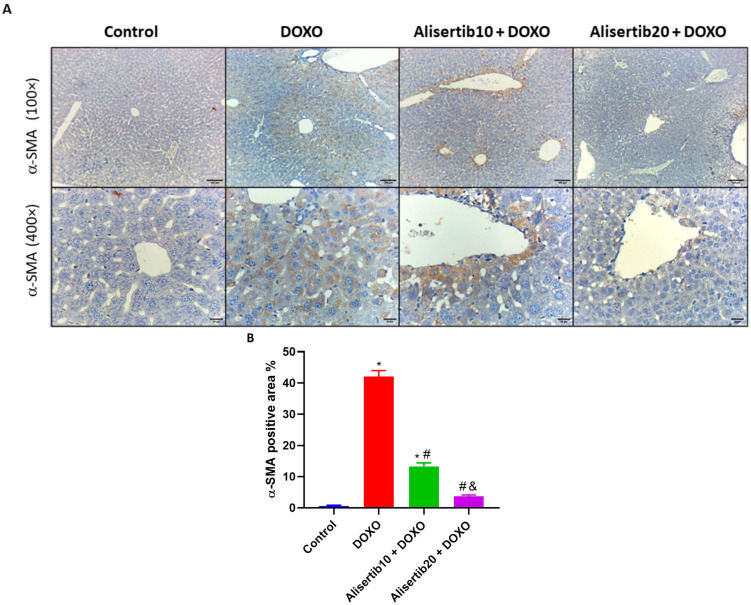
(**A**) Representative micrographs of immuno-stained liver tissue sections demonstrating the effect of alisertib (10 and 20 mg/kg) on alpha smooth muscle actin (α-SMA) expression in doxorubicin (DOXO)-intoxicated mice (upper panel: ×10, bar = 100 µm; lower panel: ×40, bar = 20 µm). The control group shows normal hepatic architecture with no α-SMA immunoreactivity. The DOXO group shows intense immunoreactivity in perivascular hepatocytes. The alisertib10 + DOXO group shows intense immunoreactivity in some perivascular hepatocytes. The alisertib20 + DOXO group shows minimal immunoreactivity in a few perivascular hepatocytes. (**B**) Corresponding percentages of α-SMA immunoexpression-positive areas. Data are expressed as means ± SEs. *, #, and & indicate significant differences compared to the control, DOXO, and alisertib10 + DOXO groups, respectively, using one-way ANOVA followed by the Tukey–Kramer multiple-comparisons post hoc test.

Furthermore, the immunoexpression of α-SMA in the liver was addressed (Figure 6A). Our findings showed that the DOXO group had intense cytoplasmic α-SMA immunoreactivity in perivascular hepatocytes, while the control group had nearly negative immunoreactivity. Otherwise, the alisertib10 + DOXO group demonstrated intense cytoplasmic α-SMA immunoreactivity in some perivascular hepatocytes, and the alisertib20 + DOXO group showed minimal cytoplasmic α-SMA immunoreactivity in a few perivascular hepatocytes. Additionally, the percentage of α-SMA-positive area was measured (Figure 6B). The DOXO challenge significantly (*p* < 0.001) elevated hepatic α-SMA expression levels by about 64.7-fold, compared to the control group. Meanwhile, alisertib administration (10 mg/kg) with DOXO significantly (*p* < 0.001) reduced the elevated α-SMA levels by about 68.4% when compared to the DOXO group, and these levels were further repressed when alisertib (20 mg/kg) was administered with DOXO by about 91% compared to DOXO alone.

### 2.6. Effect of Alisertib on DOXO-Induced Elevation of HIF-1α and VEGF-A Hepatic Expression

**Figure 7:**
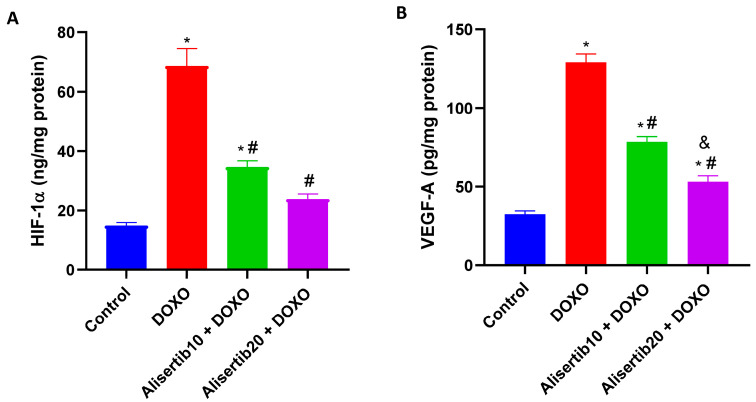
Effect of alisertib (10 and 20 mg/kg) on hepatic levels of (**A**) hypoxia-inducible factor (HIF)-1α and (**B**) vascular endothelial growth factor (VEGF)-A in doxorubicin (DOXO)-intoxicated mice. Data are expressed as means ± SEs (n = 6). *, #, and & indicate significant differences compared to the control, DOXO, and alisertib10 + DOXO groups, respectively, using one-way ANOVA followed by the Tukey–Kramer multiple-comparisons post hoc test.

The DOXO challenge caused a significant (*p* < 0.001) increase in the hepatic levels of HIF-1α (Figure 7A) and VEGF-A (Figure 7B) by about 4.6- and 3.9-fold, respectively, compared to the control group. Meanwhile, alisertib (10 and 20 mg/kg)-treated groups demonstrated significantly (*p* < 0.001) lower levels of HIF-1α (by about 49.7% and 65.3%, respectively) and VEGF-A (by about 39.2% and 59%, respectively) compared to the DOXO group. Notably the effect of the higher dose of alisertib was significantly (*p* < 0.01) higher than the lower one on hepatic VEGF-A levels.

### 2.7. Effect of Alisertib on DOXO-Induced Hepatic Activation of STAT3 and PERK

**Figure 8:**
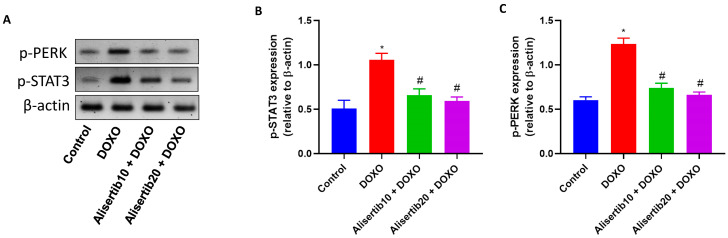
(**A**) Representative Western blot images demonstrating the effect of alisertib (10 and 20 mg/kg) on hepatic phosphorylated signal transducer and activator of transcription 3 (p-STAT3) and phosphorylated protein kinase RNA-like endoplasmic reticulum kinase (p-PERK). (**B**,**C**) Corresponding hepatic relative expression levels of p-STAT3 and p-PERK. Data are expressed as means ± SEs (n = 3). * and # indicate significant differences compared to the control and DOXO groups, respectively, using one-way ANOVA followed by the Tukey–Kramer multiple-comparisons post hoc test.

The active STAT3 and PERK protein expression was evaluated by Western blot analysis (Figure 8A and Figures S1–S3). Our data revealed that DOXO injection strongly elevated both active STAT3 and PERK protein expression; however, this elevated expression was diminished upon treatment with alisertib (10 mg/kg) and was further suppressed by alisertib (20 mg/kg). The effect of alisertib on STAT3 and PERK protein expression was additionally illustrated in Figure 8B and Figure 8C, respectively, which show significant (*p* < 0.001) increases in active STAT3 and PERK expression in the liver following the DOXO challenge by about 2.1- and 2-fold, respectively, compared to the control group. Alternatively, treatment with alisertib (10 and 20 mg/kg) with DOXO significantly suppressed the hepatic levels of active STAT3 (by about 37.6% and 44%, respectively) and PERK (by about 40.1% and 46.4%, respectively) compared to DOXO alone.

## 3. Discussion

DOXO-associated hepatotoxicity has flawed its clinical use in cancer patients. The current study explored the potential protective effect of the AURKA inhibitor alisertib on DOXO-induced hepatotoxicity and explained how it could work. Our data indicated potent hepatoprotective activities of alisertib that mitigated DOXO-induced hepatotoxicity. These activities may be mediated via targeting the IL-17A/NF-κB and IL-17A/STAT3/HIF-1α/VEGF-A signaling pathways, attenuating oxidative stress, inflammation, ER stress, and fibrosis.

DOXO-induced oxidative stress is linked to its chemical structure, which mediates ROS generation, inducing redox imbalance and subsequent hepatocyte injury [18,19]. Herein, DOXO-induced hepatocyte injury was represented by markedly elevated serum levels of the liver enzymes AST, ALT, and LDH, which were accompanied by high levels of the lipid peroxidation biomarker MDA and low levels of hepatic TAC. On the other hand, alisertib strongly protected hepatocytes from injury, reducing liver enzyme and MDA levels and elevating TAC levels. These effects were reflected in the histopathological findings that revealed repressed hepatic necroinflammation upon alisertib intervention, implying an ability of alisertib to attenuate DOXO-induced liver injury.

Oxidative stress and necrotic cell death give a spark to the initiation of an inflammatory response, which is a crucial mediator of the hepatotoxic effect of DOXO [20]. Among the key players during liver inflammation is IL-17A, which aggravates liver injury [21,22]. IL-17A stimulates downstream nuclear translocation and activation of the master transcription factor NF-κB, which in turn is responsible for the production of various inflammatory cytokines and plays a crucial role in DOXO-induced hepatotoxicity [23,24,25]. Consistently, our results show that the DOXO challenge provoked the IL-17A/NF-κB axis, which was effectively suppressed upon alisertib intervention.

IL-17A also plays a role in the activation of the transcription factor STAT3, which stimulates HIF-1α expression and accelerates the progression of liver injury to fibrosis [26,27,28]. STAT3/HIF-1α has been linked to enhanced expression of pro-fibrotic genes like TGF-β1 and VEGF-A, the major factor in angiogenesis [29]. TGF-β1 and VEGF-A exacerbate liver injury and promote fibrosis [30,31]. In the present study, the DOXO challenge enhanced the activity of STAT3 and upregulated HIF-1α, TGF-β1, and VEGF-A, as well as α-SMA expression, in the liver, which were counteracted by alisertib intervention. This indicates that alisertib not only exerts anti-inflammatory but also anti-fibrotic effects on DOXO-induced hepatotoxicity.

Consistent with our results, Kabel et al. (2018) showed upregulated TGF-β1 and STAT3 expression in DOXO-induced hepatotoxicity in mice [5]. Moreover, the selective inhibition of AURKA has significantly reduced TGF-β levels and attenuated renal fibrosis [32] and primary myelofibrosis [33] in vivo in mouse models. The role of AURKA in STAT3 activity was previously reported, and alisertib has shown an inhibitory effect on STAT3 activation [13,34,35]. In addition, AURKA inhibition has shown a negative effect on VEGF transcription and release in vitro in human umbilical vein endothelial cells [36] as well as HIF-1α expression in vitro in metastatic breast cancer [37].

The endoplasmic reticulum (ER) stress sensor PERK has also been associated with exacerbated liver inflammation and fibrosis. Upon activation, PERK boosts NF-κB signaling pathways and promotes the expression of pro-inflammatory and profibrogenic factors [38,39,40]. In addition, PERK is linked to angiogenesis and upregulated VEGF expression, leading to excessive tissue deposition and fibrosis [41,42]. In the current study, alisertib intervention markedly inhibited DOXO-induced PERK activation.

Importantly, while previous studies have established that DOXO induces oxidative stress and NF-κB activation in liver injury, our work is the first to demonstrate that selective AURKA inhibition with alisertib can concurrently suppress the IL-17A/NF-κB and IL-17A/STAT3/HIF-1α/TGF-β1/VEGF-A signaling pathways. This unique dual pathway modulation uncovers a novel mechanism for mitigating anthracycline hepatotoxicity beyond the classical NF-κB axis.

From a translational perspective, these results suggest that inhibition of AURKA could be used as an adjuvant strategy in anthracycline-based chemotherapy, not only to enhance the antitumor activity but also to attenuate off-target organ toxicities. Future preclinical research should investigate alisertib with DOXO combination regimens to assess synergistic therapeutic effect and safety profile using tumor-bearing models.

It also remains to be clarified whether the hepatoprotective effects of AURKA inhibition occur through direct modulation of hepatic signaling or indirectly via upstream regulators of oxidative stress and inflammation. Prior evidence indicates AURKA can directly interact with STAT3 and NF-κB nodes [43,44], yet the attenuation of IL-17A-driven responses observed here may also involve indirect crosstalk.

However, alisertib has high selectivity against AURKA, and it is worth mentioning that previous studies have reported some activity against AURKB and other kinases at higher concentrations [45,46]. Consequently, the observed hepatoprotective benefits may partially be associated with off-target modulation of related pathways. Future mechanistic studies using genetic AURKA knockdown are required to distinguish between direct and indirect effects and confirm AURKA’s direct role in DOXO-induced hepatotoxicity. Moreover, future studies using both sexes and chronic dosing models are recommended.

## 4. Materials and Methods

### 4.1. Drugs and Chemicals

DOXO was purchased as an injectable solution (50 mg/25 mL; Ebewe pharma, Unterach, Austria). Alisertib was purchased as a pure powder from DC Chemicals (Shanghai, China, Cat. No: DC2016, Formula: C27H20CLFN4O4, CAS: 1028486-01-2, IC50: 1.2 nM) and prepared as a fresh solution (1.5 mg/mL) in vehicle (5% DMSO and 95% normal saline). All other chemicals used in this study were of high analytical grade.

### 4.2. Animals

Adult male *BALB/c* mice weighing 20–25 g were obtained from VACSERA (Giza, Egypt) and housed in a controlled environment maintained at 25 °C under a 12 h/12 h light/dark cycle with free access to food and water. All care and experimental protocols were reviewed and approved by the Mansoura University Animal Care and Use Committee (MU-ACUC), Mansoura University, Egypt (PHARM.MS.23.10.24).

### 4.3. Experimental Design

The mice were randomly divided into the following five groups (six mice each): Group I (Control) received the alisertib vehicle (13.3 mL/kg) for five consecutive days; Group II (Alisertib) was intraperitoneally (i.p.) injected with alisertib (20 mg/kg, 13.3 mL/kg) daily for five consecutive days and normal saline once on day two; Group III (DOXO) was injected with the alisertib vehicle (13.3 mL/kg) daily for five consecutive days and received DOXO (20 mg/kg, 12.5 mL/kg, i.p.) once on day two (DOXO challenge); Group IV (Alisertib10 + DOXO) received alisertib (10 mg/kg, 6.65 mL/kg, i.p.) daily for five consecutive days and was challenged with DOXO as described above; Group V (Alisertib20 + DOXO) received alisertib (20 mg/kg, 13.3 mL/kg, i.p.) daily for five consecutive days and was challenged with DOXO as described above. The doses were selected based on our preliminary trials and in the light of previous studies on alisertib [32,45,47] and DOXO [48].

### 4.4. Sample Collection and Preparation

Twenty-four hours after administration of the last dose of alisertib, the mice were anesthetized with thiopental (70 mg/kg, i.p.) and blood samples were collected from the heart and then centrifuged at 1000× *g* for 10 min at 4 °C to separate serum samples, which were used for further biochemical analysis. The right median lobe of the liver was isolated and fixed in neutral buffered formalin (10% *v*/*v*) to prepare paraffin blocks for subsequent histopathological and immunohistochemical analyses. On the other hand, the left median lobe was isolated and cut into two parts: the first part was stored at −80 °C for Western blot analysis, whereas the second part was homogenized in phosphate-buffered saline (10% *w*/*v*) and then centrifuged at 3000× *g* for 20 min at 4 °C to collect the supernatant (homogenate), which was kept at −80 °C for further measurements.

### 4.5. Assessment of Liver Function

Alanine aminotransferase (ALT) and aspartate aminotransferase (AST) enzyme activities in the serum were determined using corresponding specific kits (Agappe Diagnostics Ltd., Kochi, India) and following the manufacturer’s instructions. In addition, serum lactate dehydrogenase (LDH) activity was determined using a specific kit (Swemed diagnostics, Bengaluru, India).

### 4.6. Assessment of Hepatic Redox Status

The lipid peroxidation biomarker malondialdehyde (MDA) and the total antioxidant capacity (TAC) were determined in the liver homogenate samples using specific corresponding colorimetric kits (Bio-diagnostic, Giza, Egypt), following the manufacturer’s directions. MDA was assessed by measuring thiobarbituric acid reactive substances according to the method of Ohkawa et al. [49]. Regarding TAC, it was detected based on the reduction of 3,5-dichlorobenzene sulfonate to a stable colored product by antioxidants in the homogenate.

### 4.7. Histopathological Assessment of the Liver

Liver sections (4 μm in thickness) were prepared from the paraffin blocks, mounted on slides, and stained with hematoxylin and eosin (H&E) and Masson’s trichrome (MT) to allocate necroinflammatory and fibrotic changes, respectively. The slides were photographed using a digital eyepiece (MVV5000CL, Hangzhou Future Optics Sci & Tech Co., Ltd., Hangzhou, China, Future WinJoe software, version 1.6.5) installed on a microscope (MEIJI MX5200L, Saitama, Japan) using 10× and 40× objectives. Histopathological lesions were blindly scored as a summation of sinusoidal congestion, hepatocyte necrosis, and ballooning degeneration scores, each graded from 0 (no damage) to 4 (severe damage), as previously described by Suzuki et al. [50]. At least ten fields (10×) were reviewed for each slide. For measurement of fibrotic surface area percentage, the resulting 10× images were analyzed on an Intel^®^ Core I7^®^-based computer using Fiji ImageJ software (version 1.51r; NIH, Bethesda, MD, USA) and the color deconvolution 2 plugin (histological dyes digital separation). Five random fields sized 200 × 200 µm from each slide were analyzed.

### 4.8. Immunohistochemistry

Immunohistochemical staining was conducted to assess the hepatic expression of the nuclear factor kappa B (NF-κB) p65 and alpha smooth muscle actin (α-SMA) using specific corresponding primary antibodies [Cat No. A2547, ABclonal, Woburn, MA, USA (dilution 1:200) and Cat No. GB111364, Servicebio, Wuhan, China (dilution 1:1000), respectively]. Slides were photographed using the MVV5000CL digital eyepiece installed on the MEIJI MX5200L light microscope, a 0.5 photo adaptor, and Future WinJoe software, using 10× and 40× objectives. The resulting 10× images were analyzed on an Intel^®^ Core I7^®^-based computer using Fiji ImageJ software (version 1.51r; NIH, Bethesda, MD, USA). For measuring staining surface area, the color deconvolution 2 plugin was used (histological dyes digital separation). This provided three independent digital images (hematoxylin, DAB, and a complementary image). DAB digital images were then used for quantification. Five random fields sized 200 × 200 µm from each slide were analyzed.

### 4.9. ELISA Measurements

NF-κB p65, interleukin (IL)-17A, hypoxia-inducible factor (HIF)-1α, transforming growth factor beta 1 (TGF-β1), and vascular endothelial growth factor (VEGF)-A levels were estimated in liver homogenates using specific corresponding ELISA kits (MyBiosource, San Diego, CA, USA, for NF-κB p65 and R&D Systems Inc., Minneapolis, MN, USA, for IL-17A, HIF-1α, TGF-β1, and VEGF-A), according to the manufacturers’ instructions.

### 4.10. Western Blot Analysis

In brief, total proteins were extracted from liver tissue samples using RIPA lysis buffer, and their concentrations were determined using the Bradford Protein Assay Kit (Thermofisher, Waltham, MA, USA). Each sample was loaded with 2× Laemmli sample buffer and boiled at 95 °C for 5 min to ensure denaturation before loading on polyacrylamide gel electrophoresis. Equivalent amounts of each sample (20 µg of proteins) were loaded on the gel. The gel was run for 20 min at 50 volt and then increased to 100–150 volt for about 1 h. The gel was then sandwiched as follows: filter paper, PVDF membrane, gel, and filter paper. The sandwich was placed in a transfer tank with buffer and run for 7 min at 25 volts. The membrane was blocked using tris-buffered saline with Tween 20 buffer (TBST) and 3% bovine serum albumin for 1 h. Primary antibodies were used against phosphorylated ER-induced protein kinase RNA-like endoplasmic reticulum kinase (p-PERK) [Thr982, Cat No. PA5-40294, Thermofisher, Waltham, MA, USA (dilution 1:2000)] and signal transducer and activator of transcription 3 (p-STAT3) [Ser727, PA5-17876, Thermofisher, Waltham, MA, USA (dilution 1:1000)] and incubated overnight against the blotted target protein at 4 °C. The blot was then rinsed with TBST. The HRP-conjugated secondary antibody (Goat anti-rabbit IgG-HRP) was incubated for 1 h at room temperature, and the blot was rinsed with TBST. The chemiluminescent substrate (Cat No. 170-5060, Clarity^TM^ Western ECL substrate, Bio-Rad, Hercules, CA, USA) was utilized for visualization, using a CCD camera-based imager. Image analysis software was used to read the band intensity of the target proteins and the reference protein β-actin.

### 4.11. Statistical Analysis

Statistical analyses were performed using GraphPad Prism V 8.0 (GraphPad Software Inc., San Diego, CA, USA). Parametric data were analyzed using the one-way analysis of variance (ANOVA) followed by Tukey–Kramer’s multiple-comparisons post hoc test and expressed as means ± standard errors (SEs). Otherwise, non-parametric scoring data were analyzed using the Kruskal–Wallis test followed by Dunn’s tests for pairwise comparisons and expressed as medians and interquartile ranges. A *p* value less than 0.05 was set as statistically significant.

## 5. Conclusions

In conclusion, this study provides molecular insights into the hepatoprotective effect of the AURKA-selective inhibitor alisertib against DOXO-induced hepatotoxicity in mice. This effect is mainly mediated via suppressing the hepatic IL-17A/NF-κB and IL-17A/STAT3/HIF-1α/VEGF-A intercalated signaling pathways, preserving liver histological structure and function, attenuating oxidative stress, suppressing inflammation, repressing ER stress, and inhibiting fibrosis.

While DOXO-induced oxidative stress and NF-κB activation are well documented, our findings are the first to demonstrate that selective AURKA inhibition can simultaneously downregulate the IL-17A-driven STAT3/HIF-1α/TGF-β1/VEGF-A axis in DOXO-induced liver injury. This dual pathway modulation reveals a novel therapeutic mechanism for mitigating anthracycline hepatotoxicity beyond the classical NF-κB pathway. These findings open new avenues for therapeutic interventions targeting AURKA, not only in DOXO-induced hepatotoxicity but also in its other associated toxicities, as well as different anticancer-induced toxicities.

## Data Availability

The original contributions presented in this study are included in the article/Supplementary Materials. Further inquiries can be directed to the corresponding authors.

## Supplementary Materials

The following supporting information can be downloaded at: https://www.mdpi.com/article/10.3390/ph18081201/s1, Figures S1–S3: Uncropped raw Western blot images for β-actin, p-STAT3, and p-PERK, respectively.
