# Supplementary material for: The AURKA-Selective Inhibitor Alisertib Attenuates Doxorubicin-Induced Hepatotoxicity in Mice via Modulation of IL-17A/NF-κB and STAT3 Signaling Pathways
Source: Pharmaceuticals (Basel). 2025 Aug 14;18(8):1201. doi: 10.3390/ph18081201 (PMC12389450; doi:10.3390/ph18081201)
Supplement: Supplementary file 1 [file pharmaceuticals-18-01201-s001.zip › pharmaceuticals-3769624-supplementary.pdf]

The uncropped raw Western blot images

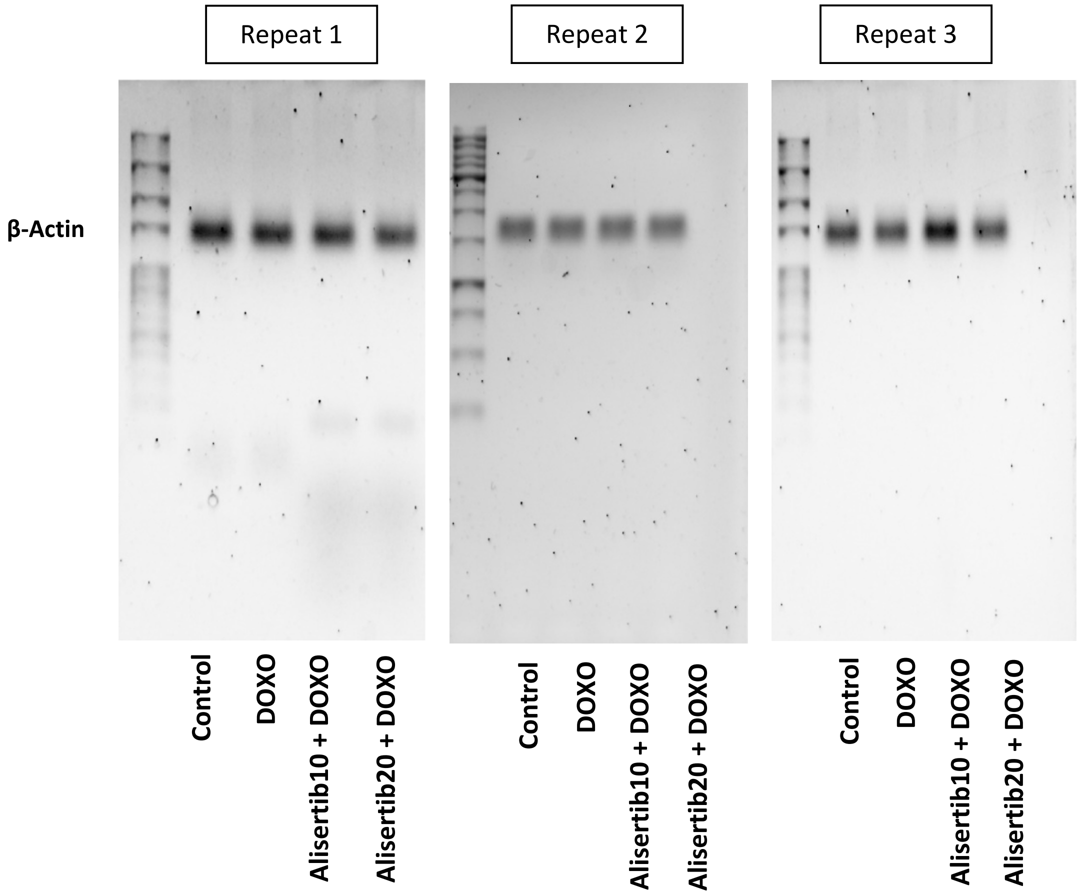

Figure S1: Uncropped raw Western blot images for β-actin.

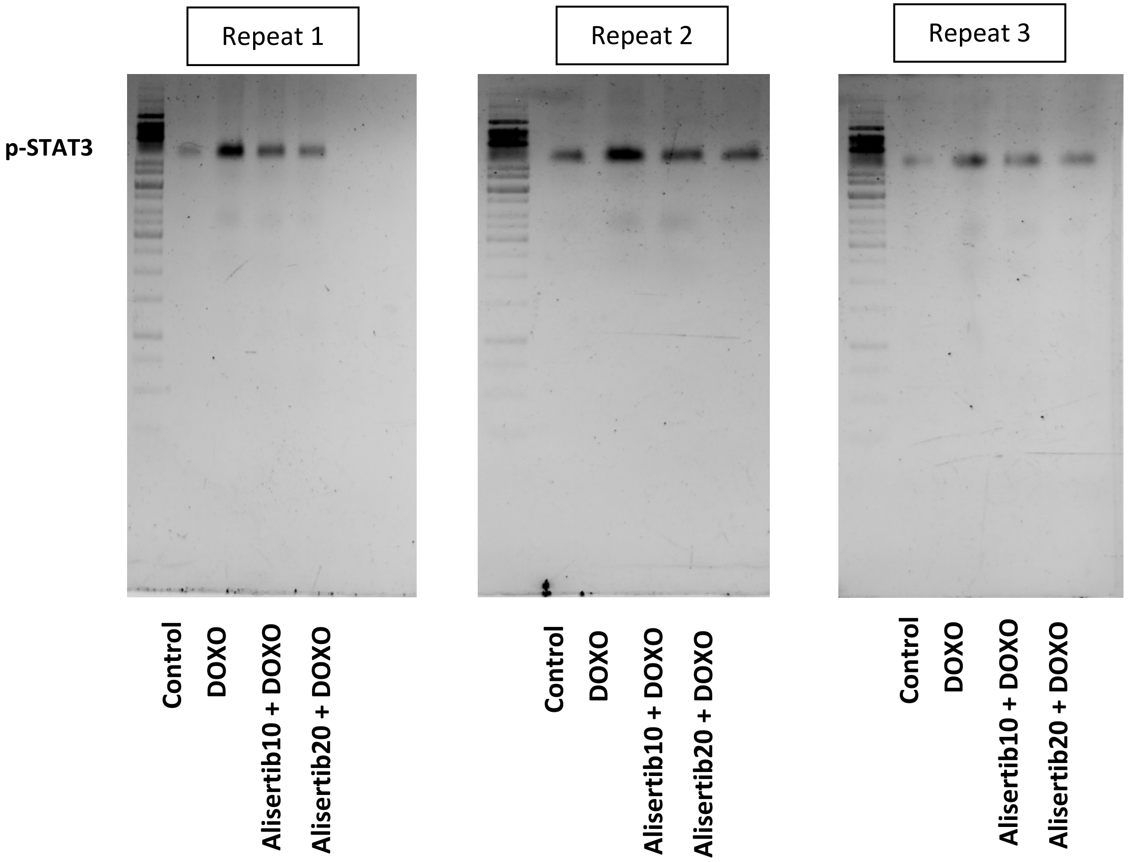

Figure S2: Uncropped raw Western blot images for p-STAT3.

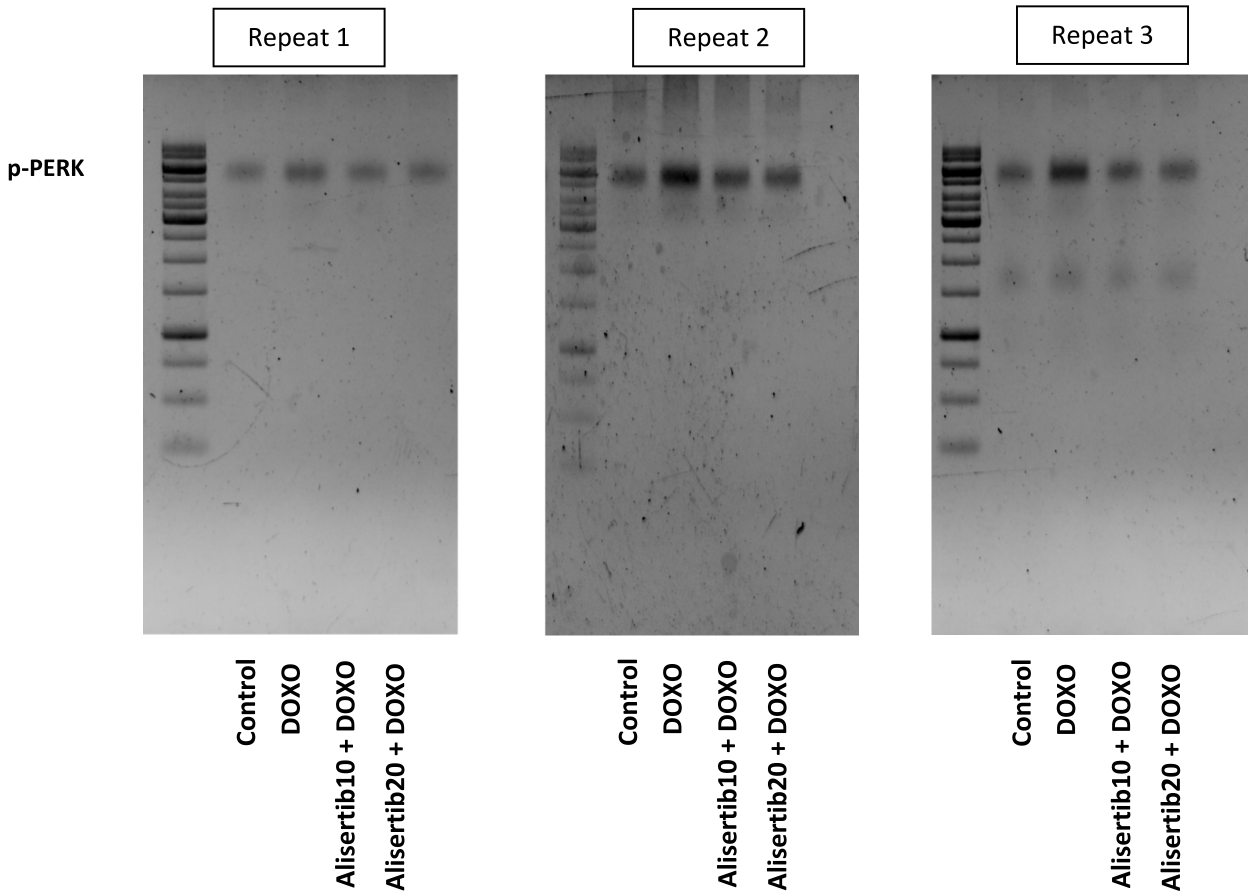

Figure S3: Uncropped raw Western blot images for p-PERK.
